# Predicting red breast syndrome after ADM-assisted breast reconstruction: An exploratory scoring model using routine hematologic markers

**DOI:** 10.1371/journal.pone.0359415

**Published:** 2026-10-07

**Authors:** Dong Hun Lee, Kyung Do Byun, Tae Hyun Kim, Jin Lee, Jiyoung Yun, Hee Yeon Kim

**Affiliations:** 1 Department of Plastic Surgery, Busan Paik Hospital, Inje University College of Medicine, Busan, Republic of Korea; 2 Department of Surgery, Busan Paik Hospital, Inje University College of Medicine, Busan, Republic of Korea; 3 Department of Neurosurgery, Busan Paik Hospital, Busan, Republic of Korea; Versiti Blood Research Institute, UNITED STATES OF AMERICA

## Abstract

**Background:**

Red breast syndrome (RBS) is a challenging complication of acellular dermal matrix (ADM)-assisted breast reconstruction. Its incidence and hematologic predictors in patients receiving neoadjuvant chemotherapy (NAC) remain poorly characterized.

**Methods:**

This retrospective cohort study included 70 patients who underwent skin-sparing mastectomy with immediate implant-based reconstruction following NAC (January 2011–May 2025) at a single tertiary institution. Patients were stratified into ADM (n = 47) and Non-ADM (n = 23) groups. Within the ADM group, Firth penalized logistic regression with backward elimination identified preoperative variables independently associated with RBS. Optimal cutoffs were determined by the Youden Index. A composite hematologic scoring model (WBC + PLT + Ki-67 High, cutoff ≥2) was developed and internally validated using bootstrap resampling (2,000 iterations).

**Results:**

RBS occurred in 31.9% (15/47) of ADM patients versus 0% in Non-ADM patients (*p* = 0.001). The final model identified WBC (OR 1.235, 95% CI 1.066–1.502, *p* = 0.004), PLT (OR 1.013, 95% CI 1.001–1.028, *p* = 0.037), and Ki-67 High (OR 8.975, 95% CI 1.356–155.173, *p* = 0.020) as independently associated with RBS. Individual biomarkers showed modest discrimination (AUC 0.605–0.641, all *p* > 0.10), while the composite score achieved AUC = 0.816 (*p* < 0.001; sensitivity 86.7%, specificity 71.9%, NPV 92.0%). Bootstrap-corrected AUC was 0.817 (95% CI 0.636–0.959; optimism=−0.001). No significant temporal trend in RBS incidence was observed (Cochran–Armitage, *p* = 0.527).

**Conclusions:**

RBS incidence is substantially elevated in post-NAC ADM patients. This exploratory, single-center study develops a hypothesis-generating combination score for preoperative RBS risk stratification using routine laboratory and pathology assessment. External validation in prospective multi-institutional cohorts is required before clinical application.

## Introduction

Neoadjuvant chemotherapy (NAC) has become an established treatment for locally advanced breast cancer, offering tumor downstaging and increased breast-conserving surgery rates [1,2]. For patients requiring mastectomy following NAC, immediate breast reconstruction with acellular dermal matrix (ADM)-assisted implant-based techniques has gained popularity due to improved cosmetic outcomes and reduced capsular contracture rates [3–6].

Red breast syndrome (RBS), characterized by persistent erythema, inflammation, and warmth without frank infection, represents a challenging complication following ADM-assisted reconstruction [7,8]. RBS may necessitate prolonged antibiotic therapy, delayed adjuvant treatments, or implant removal, compromising aesthetic results and patient satisfaction [9]. The reported incidence of RBS varies considerably across contexts: 5–10% in breast-conserving surgery with diced ADM [10], and 8–24% (typically 10–15%) in mastectomy with ADM-assisted reconstruction [7,10,11], with a recent systematic review reporting a weighted incidence of 3.22% in series published after 2017 [11]. This variation reflects differences in patient selection, surgical techniques, ADM products, and diagnostic criteria [12].

The pathophysiology of RBS remains incompletely understood, involving immune-mediated inflammation, altered wound healing, and potential subclinical biofilm formation [8,13]. Proposed mechanisms include endotoxin contamination, preservative reactions, neovascularization processes, and delayed hypersensitivity responses [14–16]. Identified risk factors include smoking, diabetes, obesity, and radiation therapy [11,12,17]. However, the role of systemic hematologic parameters, particularly in the post-NAC setting, has received limited attention.

NAC significantly impacts hematologic profiles, including white blood cell counts, platelet levels, and hemoglobin concentrations [18]. These chemotherapy-induced alterations may compromise immune homeostasis and tissue repair capacity at the time of surgery, potentially predisposing the reconstructed breast to exaggerated inflammatory responses [19–21]. The post-NAC patient undergoing immediate ADM-assisted reconstruction therefore represents a uniquely vulnerable cohort: the immunomodulatory effects of chemotherapy, superimposed on ADM implantation into previously treated tissue, may create a microenvironment particularly susceptible to RBS.

Emerging evidence supports a role for preoperative hematologic status in determining post-surgical inflammatory outcomes. Platelets serve as key regulators of the post-surgical inflammatory milieu through the release of platelet-derived growth factor (PDGF), transforming growth factor-β (TGF-β), and pro-inflammatory cytokines [22–24]. Elevated preoperative platelet counts have been independently associated with increased complications in breast reconstruction [25]. Elevated white blood cell counts may similarly reflect a pre-existing subclinical inflammatory state that renders ADM-implanted tissue susceptible to immune-mediated injury. In addition to hematologic parameters, tumor proliferative activity, commonly assessed by Ki-67 immunohistochemical expression, has been associated with increased stromal tumor-infiltrating lymphocyte density and a more immunologically active tumor microenvironment [26], raising the possibility that tumor biology may also inform host inflammatory susceptibility in the post-NAC surgical setting. Despite this biological plausibility, the predictive value of these readily available preoperative complete blood count (CBC) and pathology parameters for RBS has not been systematically evaluated, and no study has examined whether their combination yields clinically meaningful risk stratification.

Therefore, we aimed to: (1) determine RBS incidence in patients undergoing skin-sparing mastectomy with ADM-assisted immediate reconstruction following NAC and compare rates with the Non-ADM cohort; (2) identify independent preoperative predictors of RBS; (3) establish optimal cutoff values for each significant hematologic and pathologic parameter; and (4) develop and internally validate a composite scoring model—integrating white blood cell count (WBC), platelet count (PLT), and Ki-67 expression—to assess whether their combination provides superior discriminatory ability for RBS risk stratification compared to individual biomarkers alone.

## Methods

### Study design and patient selection

This retrospective cohort study was conducted at a single tertiary care institution. We identified all consecutive patients who underwent skin-sparing mastectomy with immediate implant-based breast reconstruction following completion of NAC between January 2011 and May 2025. Patient-level data were accessed from electronic medical records for research purposes on January 20, 2026. The study protocol was approved by the Institutional Review Board of Inje University Busan Paik Hospital (IRB No. 2026-01-031), and the requirement for informed consent was waived due to the retrospective nature of the study.

The IRB granted this waiver on the grounds that: (1) the retrospective nature of the study rendered prospective consent collection impracticable; (2) the research posed no more than minimal risk to participants; and (3) the waiver would not adversely affect the rights and welfare of the participants. All data were handled in accordance with the ethical standards of the institution and the 1964 Declaration of Helsinki and its later amendments. Authors did not have access to information that could identify individual participants, as all unnecessary personal identifiers were removed prior to data collection and data were de-identified before statistical analysis.

Inclusion criteria were: (1) histologically confirmed invasive breast cancer or ductal carcinoma in situ, (2) completion of NAC according to standard institutional protocols, (3) skin-sparing mastectomy with immediate implant-based reconstruction (tissue expander or permanent implant), and (4) availability of complete preoperative hematologic laboratory data obtained within 2 weeks prior to surgery. Exclusion criteria included: (1) previous breast surgery on the ipsilateral side except for diagnostic biopsy, (2) prior breast radiation therapy, (3) stage IV disease at diagnosis, (4) active infection at the time of surgery, (5) autoimmune disorders requiring immunosuppressive therapy, and (6) incomplete medical records or loss to follow-up within 6 months postoperatively.

After systematic re-screening of the institutional database, a total of 70 patients met the inclusion criteria and were included in the final analysis. ADM-assisted reconstruction was introduced at our institution in January 2017. Patients were stratified into two groups based on the use of ADM during reconstruction: the ADM group (n = 47) who received ADM-assisted reconstruction and the Non-ADM group (n = 23) who underwent standard implant-based reconstruction without ADM. The primary analytical cohort consisted of the ADM group, within which patients were further categorized based on the development of RBS during the follow-up period. All patients were non-smokers.

### Surgical technique

Surgery was performed 3 weeks after completion of neoadjuvant chemotherapy in all patients, per institutional protocol. Granulocyte colony-stimulating factor (G-CSF) was not administered within 3 weeks prior to surgery in any patient, per institutional chemotherapy protocol. Skin-sparing mastectomy was performed using standardized techniques with preservation of the breast skin envelope and inframammary fold. For immediate reconstruction, either a tissue expander or a permanent implant was placed in the prepectoral plane. Implants used were silicone gel devices from Mentor® (Mentor Worldwide LLC, Santa Barbara, CA, USA). In the ADM group, human-derived acellular dermal matrices were used, including MegaDerm® (L&C BIO Co., Ltd., Seongnam, Republic of Korea 16 × 16 cm), AlloDerm® (AbbVie Inc., North Chicago, IL, USA 10 × 20 cm), or CGDerm® (CG Bio Inc., Seongnam, Republic of Korea 16 × 16 cm). ADM selection was based on availability and surgeon preference. ADM sheets were soaked in sterile 0.9% sodium chloride solution for 10–15 minutes and rinsed three times prior to implantation. The implant was positioned above the pectoralis major muscle, and the ADM was draped over the implant to provide anterior coverage. The ADM was secured circumferentially to the surrounding soft tissue using interrupted absorbable 3−0 Vicryl sutures, thereby creating and reinforcing the prosthetic pocket. Before implant insertion, the pocket was irrigated with sterile saline.

### Definition of red breast syndrome

RBS was diagnosed based on pre-specified criteria adapted from published literature [7,8], given the absence of a universally accepted consensus definition: [11] (1) persistent erythema involving more than 50% of the breast skin envelope overlying the ADM, present beyond 2–3 weeks post-surgery; [11] (2) warmth on palpation without fluctuance or purulent drainage; [8] (3) symptoms lasting for at least 2 weeks despite conservative management; (4) absence of bacteriologic evidence of infection (negative wound culture, or C-reactive protein and WBC within normal limits at diagnosis); [10] and (5) clinical response to oral corticosteroid therapy (prednisolone) with or without concurrent antibiotics. All records were retrospectively reviewed by two experienced breast surgeons (T.H.K. and H.Y.K.), with inter-reviewer agreement complete across all cases (κ = 1.0); a third reviewer (J.Y.) was available for discrepancy resolution but was not required. Of 47 ADM patients, 20 were flagged as potential RBS during record review. Of these, 5 were reclassified as bacterial infection confirmed by positive wound culture and excluded from the RBS endpoint; the remaining 15 fulfilled all five pre-specified criteria and were classified as RBS.

### Data collection

Comprehensive clinical and pathological data were extracted from electronic medical records. Demographic variables included age at surgery and body mass index (BMI). Comorbidities including diabetes mellitus and hypertension were recorded. Surgical variables included implant type, operative time, estimated blood loss, and administration of adjuvant radiotherapy. Pathological features included immunohistochemical subtype, histologic grade (1–3), post-NAC pathologic T and N stage, residual cancer burden (RCB) grade (0–III), and Ki-67 proliferation index (grade 1: low, grade 2: intermediate, grade 3: high). Hematologic parameters were obtained from complete blood count with differential performed within 2 weeks prior to surgery. All patients received taxane-based neoadjuvant chemotherapy. Of the 47 ADM patients, 18 (38.3%) received an anthracycline-free regimen (TCHP: docetaxel, carboplatin, trastuzumab, and pertuzumab), and 29 (61.7%) received an anthracycline-containing regimen (AC followed by docetaxel, or weekly paclitaxel plus carboplatin followed by AC, with or without pembrolizumab).

### Statistical analysis

#### Descriptive statistics and group comparisons.

Continuous variables are presented as mean ± SD. Group comparisons used the Mann–Whitney U test for continuous variables and Fisher’s exact test or Chi-square test for categorical variables, as appropriate.

#### Firth penalized logistic regression.

Given the small number of events (15 RBS cases) and the risk of quasi-complete separation, Firth penalized logistic regression was employed throughout all analyses. All *p*-values were derived from penalized likelihood ratio tests (LRT); 95% confidence intervals were derived from profile likelihood methods. In Firth penalized regression applied to small samples, LRT *p-*values and profile likelihood CIs may diverge due to asymmetric penalized likelihood surfaces; the LRT *p*-value is the primary inferential metric in this setting, as it directly compares penalized log-likelihoods and is the recommended approach for inference in Firth regression [27,28]. Univariable analysis was performed for all variables; those with *p* < 0.20 entered the multivariable model. Backward elimination (removal *p* > 0.10 by likelihood ratio test) yielded a final 3-variable model (WBC, PLT, Ki-67 High).

#### Cutoff determination and combination score.

The predictive performance of the identified risk factors was evaluated using Receiver Operating Characteristic (ROC) curve analysis. The Area Under the Curve (AUC) was calculated to assess the discrimination ability of the individual predictors (WBC, PLT, and Ki-67 High) as well as the combined multivariable model. The AUC values range from 0.5 (no discrimination) to 1.0 (perfect discrimination), with a value greater than 0.7 generally considered acceptable discrimination. To determine the optimal cut-off values for WBC and PLT that best predict RBS, the Youden Index (J = Sensitivity + Specificity − 1) was utilized. The threshold maximizing the Youden Index was selected to provide the optimal balance between sensitivity (the ability to correctly identify patients who will develop RBS) and specificity (the ability to correctly identify patients who will not develop RBS). The performance of individual predictors at their respective cut-off values was evaluated in terms of sensitivity, specificity, positive predictive value (PPV), and negative predictive value (NPV). A combined predictive model incorporating all preoperative variables independently associated with RBS from the multivariable analysis was developed. The model’s predicted probabilities were calculated for each patient, and an optimal probability threshold was determined using ROC curve analysis. Model calibration was assessed using calibration slope and calibration-in-the-large, evaluating agreement between predicted probabilities and observed outcomes. Each parameter was binarized (Ki-67 High was inherently binary), and a composite scoring model (range 0–3) was constructed by summing the three binary flags. The optimal composite threshold (≥2) was determined by the Youden Index. AUC significance was assessed by the Hanley–McNeil method (H₀: AUC = 0.5).

#### Internal validation using bootstrap resampling.

Bootstrap internal validation was performed using 2,000 resamples generated by random sampling with replacement. For each iteration, the composite score ROC analysis was repeated. Optimism was estimated as the mean difference between training AUC (bootstrap sample) and out-of-bag test AUC. Optimism-corrected AUC = observed AUC − mean optimism. The 95% CI was derived from the 2.5th–97.5th percentiles of the bootstrap test AUC distribution.

#### Temporal trend analysis.

The Cochran–Armitage test was applied to annual RBS incidence rates and combination score positivity rates across the ADM study period (January 2017 through May 2025) to assess for significant temporal trends (*p* < 0.05).

#### Statistical software and significance.

All statistical analyses were performed using R software version 4.3.3 (R Foundation for Statistical Computing, Vienna, Austria) with the following packages: pROC and boot for ROC curve analysis and bootstrap validation, and ggplot2 for data visualization. Firth penalized logistic regression, including profile penalized likelihood confidence intervals and likelihood ratio test *p*-values, was implemented using the logistf package (v1.26.1; Heinze & Ploner), ensuring internally consistent inferential procedures. Cochran–Armitage tests used the base R stats package. Descriptive statistics and group comparisons were performed in SPSS v26.0 (IBM Corp.). All tests were two-sided; *p* < 0.05 was considered significant exce*p*t where otherwise specified (*p* < 0.20 for univariable screening; *p* < 0.10 for multivariable retention).

## Results

### Patient characteristics

The clinicopathologic characteristics of the study population are summarized in Table 1 (ADM vs. Non-ADM) and Table 2 (RBS vs. No-RBS within the ADM group). A total of 70 patients met inclusion criteria: 47 (67.1%) in the ADM group and 23 (32.9%) in the Non-ADM group. The two groups were well-balanced in most baseline characteristics (Table 1). Mean age was 48.0 ± 8.5 vs. 47.0 ± 10.7 years (p = 0.445), and mean BMI was 22.7 ± 2.8 vs. 23.0 ± 3.9 kg/m² (*p* = 0.717). Comorbidities, implant type, radiotherapy rates, operative time, blood loss, immunologic subtypes, histologic grade, ypT/N stage, and RCB distribution were similarly comparable. Among hematologic parameters, platelet counts were significantly lower in the ADM group (187.3 ± 58.1 vs. 256.6 ± 127.7 × 10³/µL; *p* = 0.006), and lymphocyte percentage was also lower (24.5 ± 13.1% vs. 29.1 ± 9.7%; *p* = 0.043). RBS occurred exclusively in the ADM group: 15 of 47 patients (31.9%) developed RBS versus 0 of 23 in the Non-ADM group (*p* = 0.001). Median follow-up in the ADM group was 30.8 months (IQR 15.3–52.3). Among RBS cases, the median time from surgery to first clinical presentation was 9.1 weeks (IQR 4.9–13.5 weeks; range 2.9–24.0 weeks). The exact date of RBS onset could not be determined retrospectively, as patients presented to the outpatient clinic after symptom development; the date of the first clinic visit at which RBS diagnostic criteria were fulfilled was used as a proxy for onset timing.

**Table 1:** Baseline clinicopathologic characteristics of breast cancer patients undergoing mastectomy after neoadjuvant chemotherapy, stratified by ADM-assisted reconstruction status.

Variables	ADM Group (N = 47)	No ADM Group (N = 23)	*p*-value
**Age (years)** ^**a**^	48.0 ± 8.5	47.0 ± 10.7	0.445
**BMI** **(kg/m²**)^**a**^	22.7 ± 2.8	23.0 ± 3.9	0.717
**Comorbidities**			
Diabetes Mellitus^b^			1.000
No	42 (89.4%)	21 (91.3%)	
Yes	5 (10.6%)	2 (8.7%)	
Hypertension^b^			0.119
No	34 (72.3%)	21 (91.3%)	
Yes	13 (27.7%)	2 (8.7%)	
**Treatment & Surgical Factors**			
Radiotherapy^b^			0.292
No	15 (31.9%)	11 (47.8%)	
Yes	32 (68.1%)	12 (52.2%)	
Implant Type^b^			0.124
Expander	29 (61.7%)	9 (39.1%)	
Permanent Implant	18 (38.3%)	14 (60.9%)	
Operation Time (min)^a^	191.6 ± 68.5	208.9 ± 50.7	0.125
Blood Loss (mL)^a^	129.8 ± 155.9	129.7 ± 121.6	0.578
**Pathologic** **Features**			
**Immunologic** **Subtype**^**b**^			0.849
Luminal A	3 (6.4%)	0 (0.0%)	
Luminal B	17 (36.2%)	7 (30.4%)	
Luminal B HER2+	9 (19.1%)	4 (17.4%)	
HER2+	10 (21.3%)	3 (13.0%)	
TNBC	8 (17.0%)	3 (13.0%)	
**Histologic** **Grade (1/2/3)**^**b**^			0.266
1	5 (10.6%)	1 (4.3%)	
2	30 (63.8%)	12 (52.2%)	
3	12 (25.5%)	10 (43.5%)	
**T** **Stage**^**b**^			0.638
0 + Tis	21 (44.7%)	13 (56.5%)	
1	18 (38.3%)	7 (30.4%)	
2	6 (12.8%)	3 (13.0%)	
3	2 (4.3%)	0 (0.0%)	
**N** **Stage**^**b**^			0.311
0	29 (61.7%)	11 (47.8%)	
≥1	18 (38.3%)	12 (52.2%)	
**RCB** **Grade**^**b**^			0.142
0 (pCR)	20 (42.6%)	7 (30.4%)	
I	5 (10.6%)	2 (8.7%)	
II	14 (29.8%)	13 (56.5%)	
III	8 (17.0%)	1 (4.3%)	
**Ki-67** **Expression**^**b**^			0.061
1 (Low)	2 (4.3%)	0 (0.0%)	
2 (Intermediate)	11 (23.4%)	4 (17.4%)	
3 (High)	34 (72.3%)	16 (69.6%)	
**Hematologic** **Profile**^**a**^			
WBC (×10³/µL)	7.7 ± 4.9	6.1 ± 4.0	0.167
Hb (g/dL)	10.9 ± 1.0	11.2 ± 1.4	0.920
Platelet (×10³/µL)	187.3 ± 58.1	256.6 ± 127.7	0.006**
Basophil (%)	0.4 ± 0.3	0.7 ± 0.7	0.133
Eosinophil (%)	0.9 ± 1.4	2.1 ± 3.6	0.140
Neutrophil (%)	62.4 ± 17.6	58.4 ± 13.4	0.118
Lymphocyte (%)	24.5 ± 13.1	29.1 ± 9.7	0.043*
**RBS** **Incidence**^**b**^			0.001
No	32 (68.1%)	23 (100.0%)	
Yes	15 (31.9%)	0 (0.0%)	

Abbreviations: ADM; acellular dermal matrix, BMI; body mass index, HER2; human epidermal growth factor receptor 2, TNBC; triple-negative breast cancer, Tis; tumor in situ, RCB; residual cancer burden, pCR; pathologic complete response, WBC, white blood cell; Hb, hemoglobin. RBS; red breast syndrome.

^a^Data are presented as mean ± standard deviation for continuous variables; *p*-values were calculated using Mann-Whitney U test.

^b^Data are presented as number (%) for categorical variables; *p*-values were calculated using Fisher’s exact test or Chi-square test, as appropriate.

*p* < 0.05 was considered statistically significant. *p < 0.05, **p < 0.01, ***p < 0.001.

**Table 2:** Baseline clinicopathologic characteristics of breast cancer patients undergoing mastectomy and ADM-assisted reconstruction after neoadjuvant chemotherapy.

Variables	RBS Group (N = 15)	No RBS Group (N = 32)	*p*-value
**Age (years)** ^**a**^	50.2 ± 9.4	46.9 ± 8.0	0.263
**BMI** **(kg/m²**)^**a**^	22.8 ± 3.0	22.6 ± 2.7	0.855
**Comorbidities**			
Diabetes Mellitus^b^			0.648
No	13 (86.7%)	29 (90.6%)	
Yes	2 (13.3%)	3 (9.4%)	
Hypertension^b^			0.728
No	10 (66.7%)	24 (75.0%)	
Yes	5 (33.3%)	8 (25.0%)	
**Treatment & Surgical Factors**			
Radiotherapy^b^			1.000
No	5 (33.3%)	10 (31.2%)	
Yes	10 (66.7%)	22 (68.8%)	
Implant Type^b^			0.524
Expander	8 (53.3%)	21 (65.6%)	
Permanent Implant	7 (46.7%)	11 (34.4%)	
Operation Time (min)^a^	175.1 ± 47.9	199.4 ± 75.8	0.321
Blood Loss (mL)^a^	89.3 ± 56.6	148.8 ± 182.9	0.982
Time to drain removal (days)	9 (9–11)	9 (9–12)	0.875
**Pathologic** **Features**			
**Immunologic** **Subtype**^**b**^			0.583
Luminal A	0 (0.0%)	3 (9.4%)	
Luminal B	7 (46.7%)	10 (31.2%)	
Luminal B HER2+	3 (20.0%)	6 (18.8%)	
HER2+	2 (13.3%)	8 (25.0%)	
TNBC	3 (20.0%)	5 (15.6%)	
**Chemotherapy regimen**			0.753
Anthracycline-containing	10 (66.7%)	19 (59.4%)	
Anthracycline-free (TCHP)	5 (33.3%)	13 (40.6%)	
**Histologic** **Grade (1/2/3)**^**b**^			0.833
1	1 (6.7%)	4 (12.5%)	
2	10 (66.7%)	20 (62.5%)	
3	4 (26.7%)	8 (25.0%)	
**T** **Stage**^**b**^			0.411
0 + Tis	5 (33.3%)	16 (50.0%)	
1	8 (53.3%)	10 (31.2%)	
2	1 (6.7%)	5 (15.6%)	
3	1 (6.7%)	1 (3.1%)	
**N** **Stage**^**b**^			0.202
0	7 (46.7%)	22 (68.8%)	
≥1	8 (53.3%)	10 (31.2%)	
**RCB** **Grade**^**b**^			0.384
0 (pCR)	5 (33.3%)	15 (46.9%)	
I	1 (6.7%)	4 (12.5%)	
II	7 (46.7%)	7 (21.9%)	
III	2 (13.3%)	6 (18.8%)	
**Ki-67** **Expression**^**b**^			0.284
1 (Low)	0 (0.0%)	2 (6.2%)	
2 (Intermediate)	2 (13.3%)	9 (28.1%)	
3 (High)	13 (86.7%)	21 (65.6%)	
**Hematologic** **Profile**^**a**^			
WBC (×10³/µL)	9.7 ± 6.6	6.7 ± 3.7	0.141
Hb (g/dL)	10.6 ± 1.1	11.1 ± 1.0	0.222
Platelet (×10³/µL)	203.7 ± 54.1	179.7 ± 59.1	0.126
Basophil (%)	0.3 ± 0.2	0.4 ± 0.3	0.145
Eosinophil (%)	0.6 ± 0.9	1.0 ± 1.6	0.927
Neutrophil (%)	66.3 ± 14.1	60.5 ± 18.9	0.288
Lymphocyte (%)	22.3 ± 11.5	25.5 ± 13.8	0.458

Abbreviations: RBS; red breast syndrome, BMI; body mass index, HER2; human epidermal growth factor receptor 2, TNBC; triple-negative breast cancer, Tis; tumor in situ, RCB; residual cancer burden, pCR; pathologic complete response, WBC, white blood cell; Hb, hemoglobin; TCHP, docetaxel, carboplatin, trastuzumab, and pertuzumab.

^a^Data are presented as mean ± standard deviation for continuous variables, except Time to drain removal, presented as median (IQR); *p*-values were calculated using Mann-Whitney U test

^b^Data are presented as number (%) for categorical variables*; p*-values were calculated using Fisher’s exact test or Chi-square test, as appropriate.

All demographic, surgical, and pathologic variables were well-balanced between RBS and No-RBS groups within the ADM cohort (Table 2). Among preoperative hematologic parameters, RBS patients had numerically higher WBC (9.7 ± 6.6 vs. 6.7 ± 3.7 × 10³/µL; *p* = 0.141), higher PLT (203.7 ± 54.1 vs. 179.7 ± 59.1 × 10³/µL; *p* = 0.126), and lower Hb (10.6 ± 1.1 vs. 11.1 ± 1.0 g/dL; *p* = 0.222), though none reached statistical significance in univariable comparison. High Ki-67 expression was numerically more common among RBS patients (86.7% vs. 65.6%; *p* = 0.284; Table 2). Among the three ADM products used, Megaderm® was most frequently employed (27/47, 57.4%), followed by AlloDerm® (11/47, 23.4%) and CGDerm® (7/47, 14.9%). RBS incidence did not differ significantly across ADM products (Megaderm® 29.6%, AlloDerm® 36.4%, CGDerm® 42.9%; *p* = 0.780; S1 Table). Among permanent implant recipients (n = 14; RBS n = 6, No-RBS n = 8), implant volume was numerically higher in RBS patients (293 ± 75 cc vs. 226 ± 51 cc; *p* = 0.081), though this difference did not reach statistical significance (S2 Table). Tissue expander patients were excluded from this analysis, as expanders do not have a fixed final volume and are instead serially filled over time to a variable target determined by clinical judgment.

### Firth penalized logistic regression

Univariable Firth logistic regression identified 7 variables with *p* < 0.20: WBC (OR 1.119 per ×10³/µL, *p* = 0.060), Hb (OR 0.671 per g/dL, *p* = 0.188), PLT (OR 1.007 per ×10³/µL, *p* = 0.192), Ki-67 High (OR 2.888, *p* = 0.148), Basophil % (OR 0.175, *p* = 0.124), ypN ≥ 1 (OR 2.429, *p* = 0.153), and Stromal TILs (OR 3.605, *p* = 0.102). ANC (OR 1.142, *p* = 0.068) also met this threshold but was excluded prior to backward elimination due to collinearity with WBC. BMI and all multi-level categorical variables (histologic grade, immunologic subtype, ypT stage, P stage, RCB grade) did not meet the *p* < 0.20 threshold when assessed by overall likelihood ratio test.

After backward elimination (removal threshold *p* > 0.10), Basophil, Stromal TILs, ypN ≥ 1, and Hb were sequentially eliminated, yielding a final 3-variable model (EPV = 5.0) that identified WBC, PLT, and Ki-67 High as preoperative variables independently associated with RBS: WBC (OR 1.235, 95% CI 1.066–1.502, *p* = 0.004), PLT (OR 1.013, 95% CI 1.001–1.028, *p* = 0.037), and Ki-67 High (OR 8.975, 95% CI 1.356–155.173, *p* = 0.020), the latter with a wide confidence interval reflecting the small number of Low/Intermediate Ki-67 cases (n = 13). The results are summarized in Table 3.

**Table 3:** Firth penalized logistic regression analysis of risk factors for red breast syndrome in breast cancer patients undergoing mastectomy and ADM-assisted reconstruction after neoadjuvant chemotherapy.

	Univariable analysis (n = 47)		Multivariable analysis (n = 47)	
**Variable**	OR [95% CI]	*p*-value	OR [95% CI]	*p*-value
**Age (years)**	1.045 [0.973-1.132]	0.231		
**BMI** **(kg/m²**)	1.028 [0.827-1.273]	0.799		
**Comorbidities**				
Diabetes Mellitus	1.561 [0.236-9.039]	0.621		
Hypertension	1.510 [0.399-5.501]	0.535		
**Implant Type**				
Expander (ref)	1.00 (reference)	–		
Permanent Implant	1.650 [0.484-5.643]	0.420		
**Treatment & Surgical Factors**				
Radiotherapy (Yes)	0.891 [0.254-3.304]	0.858		
Operation Time (min)	0.995 [0.984-1.004]	0.290		
Blood Loss (mL)	1.000 [0.004-257.800]	1.000		
**Pathologic** **Features**				
**Histologic** **Grade (1/2/3)**				
1 (ref)	1.000 (reference)	–		
2	1.537 [0.241-16.793]	0.665		
3	1.588 [0.199-19.550]	0.671		
**Ki-67 Expression**				
Low/ Intermediate(ref)	1.00 (reference)	–	1.000 (reference)	
High	2.888 [0.702-16.639]	0.148*	8.975 [1.356-155.173]	0.020*
**Immunologic** **Subtype**				
Luminal A (ref)	1.000 (reference)	–		
Luminal B	5.000 [0.393-711.213]	0.241		
Luminal B HER2+	3.769 [0.242-563.900]	0.375		
HER2+	2.059 [0.119-313.003]	0.649		
TNBC	4.455 [0.281-671.334]	0.316		
**ER** **status**				
Negative (ref)	1.000 (reference)			
Positive	1.558 [0.442-6.180]	0.497		
**PR** **status**				
Negative (ref)	1.000 (reference)	–		
Positive	1.004 [0.301-3.381]	0.995		
**c-erbB2 expression**				
0-1+ (ref)	1.000 (reference)			
2+	2.478 [0.551-11.283]	0.231		
3+	0.859 [0.234-2.946]	0.811		
**Stromal TILs(%)**	3.605 [0.776-18.981]	0.102		
**Hematologic Factors**				
WBC (×10³/µL)	1.119 [0.995-1.287]	0.060 *	1.235 [1.066–1.502]	0.004*
RBC (×10⁶/µL)	0.475 [0.114-1.743]	0.266		
Hb (g/dL)	0.671 [0.348-1.209]	0.188*		
Hct (%)	0.918 [0.763-1.090]	0.331		
Platelet (×10³/µL)	1.007 [0.997-1.018]	0.192*	1.013 [1.001–1.028]	0.037*
Basophil (%)	0.175 [0.013-1.574]	0.124*		
Eosinophil (%)	0.859 [0.485-1.302]	0.498		
**Pathologic** **Response**				
**ypT** **Stage**				
0 + Tis (ref)	1.00 (reference)	–		
1	2.429 [0.660-9.598]	0.183		
2	0.818 [0.072-5.574]	0.846		
3	3.000 [0.211-43.397]	0.388		
**ypN** **Stage**				
0 (ref)	1.00 (reference)	–		
≥1	2.429 [0.717-8.522]	0.153*		
P Stage				
0 (ref)	1.00 (reference)	–		
I	1.281[0.186-7.422]	0.787		
II	3.843[0.914-17.888]	0.066		
III	0.564[0.051-3.607]	0.563		
**RCB** **Grade**				
0 (ref)	1.00 (reference)	–		
I	0.939 [0.081-6.798]	0.953		
II	2.818 [0.706-12.103]	0.143		
III	1.084 [0.161-6.026]	0.928		

Abbreviations: OR; odds ratio, CI; confidence interval, BMI; body mass index, HER2; human epidermal growth factor receptor 2, TNBC; triple-negative breast cancer, TIS; tumor in situ, RCB; residual cancer burden, pCR; pathologic complete response, WBC, white blood cell; Hb, hemoglobin.

*Variables meeting the univariable screening threshold (*p* < 0.20). For multi-level categorical variables (Histologic Grade, Immunologic Subtype, ypT Stage, P Stage, RCB Grade), the overall likelihood ratio test *p*-value was used for screening (all overall *p* > 0.20; individual dummy-level comparisons shown above for completeness). Continuous and binary variables were screened using their individual *p*-values.All estimates were derived from Firth penalized logistic regression using the validated logistf R package (v1.26.1; Heinze & Ploner). 95% confidence intervals and p-values were computed using the penalized profile likelihood method, ensuring internal consistency between confidence intervals and hypothesis tests.Final multivariable model (backward elimination, removal threshold *p* > 0.10): WBC, PLT, and Ki-67 High were retained; Baso*p*hil, Stromal TILs, ypN ≥ 1, and Hb were sequentially eliminated (in that order).

### Cutoff determination and combination score performance

Individual biomarker performance at Youden Index cutoffs: WBC ≥ 9.45 × 10³/µL (AUC = 0.635, *p* = 0.132; sensitivity 46.7%, specificity 90.6%); PLT ≥ 177 × 10³/µL (AUC = 0.641, *p* = 0.117; sensitivity 73.3%, specificity 56.2%); Ki-67 High expression (AUC = 0.605, *p* = 0.248; sensitivity 86.7%, specificity 34.4%). None achieved statistical significance as standalone predictors (Fig 1A). The composite score (≥2 of 3 criteria: WBC, PLT, and Ki-67 High) achieved AUC = 0.816 (*p* < 0.001), a marked improvement over any individual biomarker. At the optimal threshold: sensitivity 86.7%, specificity 71.9%, PPV 59.1%, NPV 92.0% (at observed prevalence 31.9%). Among 47 ADM patients, 13 of 15 RBS patients (86.7%) were correctly identified as high-risk, while 23 of 32 Non-RBS patients (71.9%) were correctly classified as low-risk (Fig 1B).

**Fig 1:**
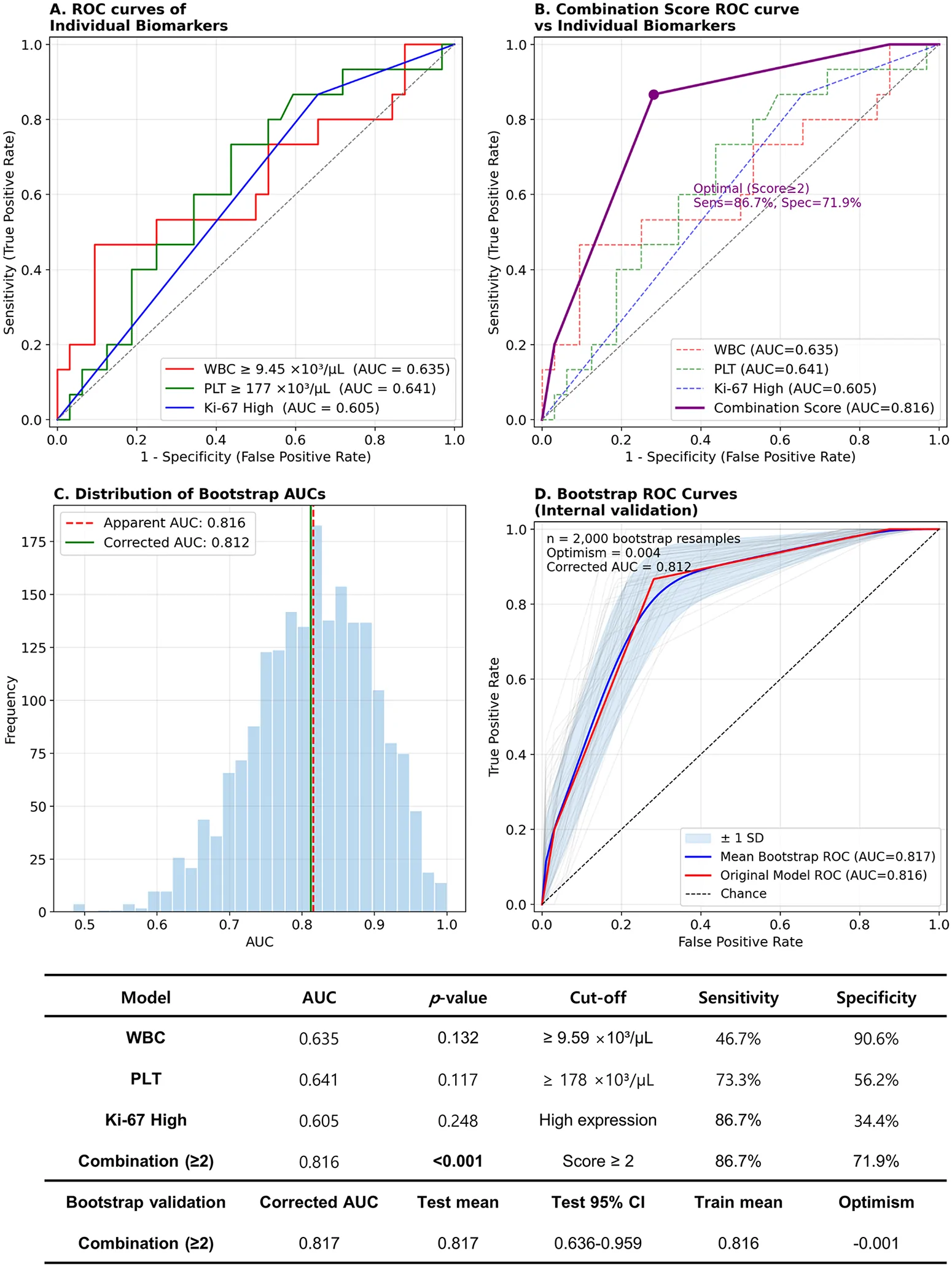
Receiver operating characteristic (ROC) curves for predicting red breast syndrome (RBS) and bootstrap internal validation in patients undergoing acellular dermal matrix-assisted immediate breast reconstruction after neoadjuvant chemotherapy. (A) ROC curves of individual biomarkers at Youden Index–derived cutoffs: WBC ≥ 9.45 × 10³/µL (AUC = 0.635), PLT ≥ 177 × 10³/µL (AUC = 0.641), and Ki-67 High expression (AUC = 0.605). None achieved statistical significance as standalone predictors (all *p* > 0.10). (B) ROC curve of the composite hematologic combination score (WBC flag + PLT flag + Ki-67 High flag, cutoff ≥2) overlaid against individual biomarker curves. The combination score achieved AUC = 0.816 (*p* < 0.001). The filled circle indicates the optimal operating point (sensitivity = 86.7%, specificity = 71.9%). (C) Distribution of bootstrap AUC values from 2,000 resamples. The red dashed vertical line indicates the apparent AUC (0.816); the green solid vertical line indicates the bootstrap-corrected AUC (0.817). The near-identical values confirm negligible overfitting (optimism=−0.001). (D) Bootstrap ROC curves from 2,000 resamples (gray), with mean bootstrap ROC (blue) and original model ROC (red) overlaid. The tight clustering of bootstrap curves around the original model confirms model stability. The embedded performance table summarizes AUC, *p*-value, cutoff, sensitivity, and specificity for each individual biomarker and the combination score, together with bootstrap validation metrics (corrected AUC = 0.817, 95% CI 0.636–0.959, test mean = 0.817, train mean = 0.816, optimism=−0.001).

### Bootstrap internal validation

Bootstrap validation (2,000 resamples) yielded mean training AUC 0.816 ± 0.058 and mean out-of-bag test AUC 0.817 ± 0.084, giving an optimism of −0.001—indicating essentially no overfitting. Optimism-corrected AUC: 0.817 (95% CI 0.636–0.959). The near-perfect overlap between training and test AUC distributions confirms that the combination score’s performance reflects genuine predictive signal (Fig 1C and 1D). Model calibration and clinical utility are summarized in S3 Table; calibration slope was 1.073 (ideal: 1.0), Brier score 0.160 (null model 0.217; scaled Brier score 0.262), and decision curve analysis demonstrated net benefit over both treat-all and treat-none strategies across threshold probabilities of 0.05–0.65.

### Temporal analysis

Cochran–Armitage trend testing across the ADM study period (2017–2025) revealed no significant temporal trend in RBS incidence rate (Z=−0.632, *p* = 0.527) nor in combination score positivity rate (Z=−1.408, *p* = 0.159). See Fig 2.

**Fig 2:**
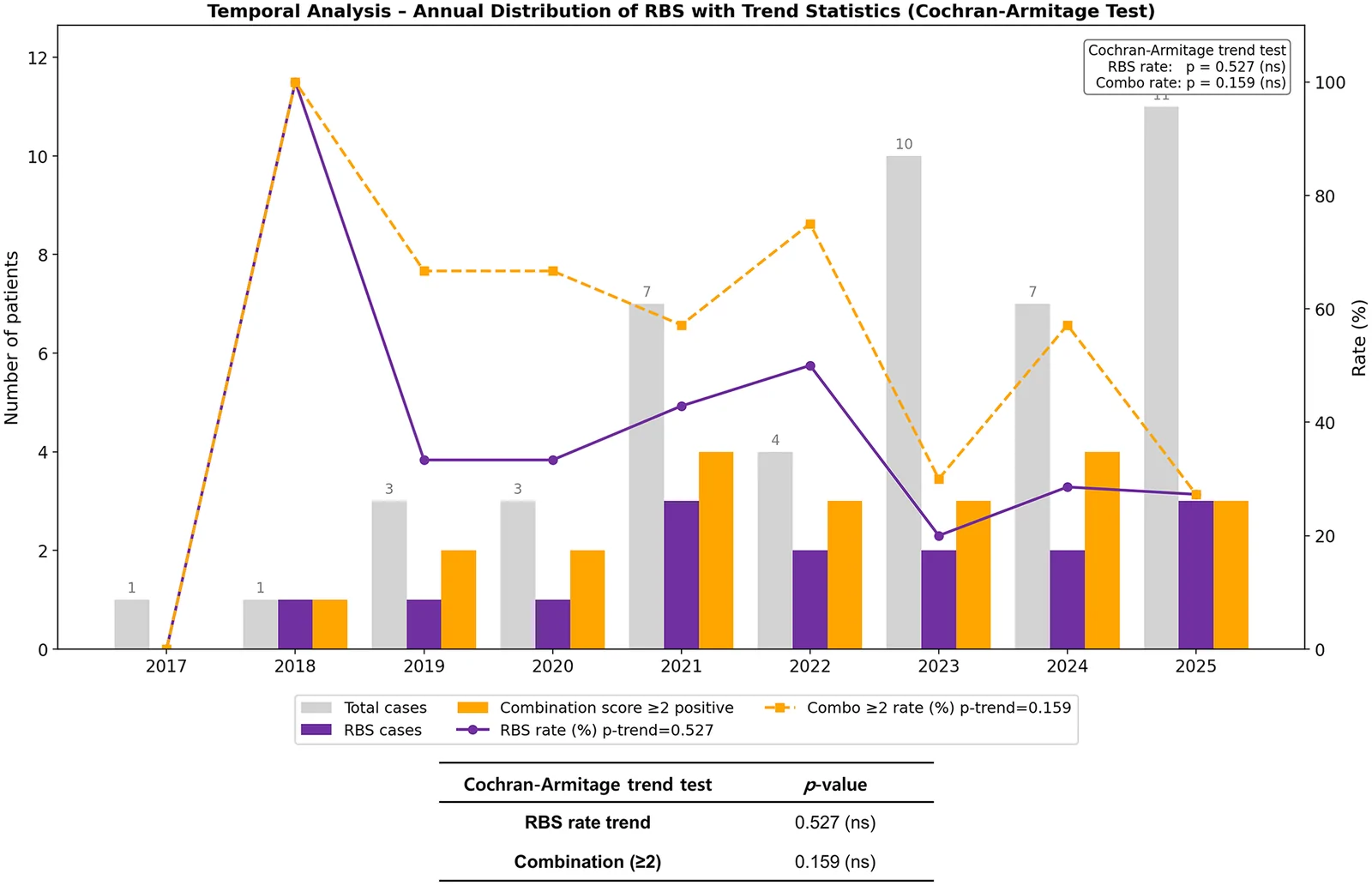
Temporal analysis of annual red breast syndrome (RBS) incidence and hematologic combination score positivity in the ADM cohort (January 2017 – May 2025).

Bar charts display annual counts of total ADM patients (gray), RBS cases (purple), and combination score ≥2 positive cases (orange). Line graphs (right axis) represent annual RBS incidence rate (%) (purple solid line) and combination score positivity rate (%) (orange dashed line). The Cochran–Armitage test for linear trend revealed no statistically significant temporal trend in RBS incidence rate (*p* = 0.527, ns) nor in combination score positivity rate (*p* = 0.159, ns), supporting the absence of systematic temporal confounding across the study period despite potential evolution in NAC regimens, ADM products, and surgical techniques over time. ns, not significant.

## Discussion

This study, to our knowledge the first to systematically examine RBS incidence and preoperative predictors in patients undergoing ADM-assisted immediate breast reconstruction following NAC, yields five principal findings: (1) a substantially elevated RBS incidence of 31.9% (15/47) in the post-NAC ADM cohort; (2) identification of three preoperative variables independently associated with RBS—elevated WBC, elevated PLT, and high Ki-67 expression; (3) a composite scoring model (WBC + PLT + Ki-67 High, cutoff ≥2) achieving AUC = 0.816 (*p* < 0.001), markedly superior to any individual biomarker (AUC 0.605–0.641, all *p* > 0.10); (4) bootstrap internal validation (2,000 resamples) confirming negligible overfitting (optimism=−0.001, corrected AUC 0.817, 95% CI 0.636–0.959); and (5) different risk factor associations compared to prior literature.

### Elevated RBS incidence in the post-NAC ADM setting

Our observed RBS incidence of 31.9% substantially exceeds the 7–9% reported in historical series and the 3.22% weighted incidence from recent studies (2017–2023) [11]. Several factors may contribute to this elevation. NAC profoundly alters hematologic and immunologic homeostasis: chemotherapy induces lymphatic injury that impairs clearance of inflammatory mediators and ADM-derived antigens [20,29], while simultaneously perturbing immune cell populations and cytokine networks [18,21]. Additionally, NAC affects tissue vascularity and wound healing at the cellular level, which may predispose the surgical site to prolonged erythema and inflammatory reactions that characterize RBS [30]. Our study is explicitly designed to characterize which preoperative patient-level factors, within a population for whom ADM use is clinically indicated, determine susceptibility to this ADM-driven inflammatory response. Temporal analysis (Cochran–Armitage *p* = 0.527) confirmed the absence of systematic temporal confounding, directly addressing concerns regarding the 9-year study period. Several additional factors merit consideration in contextualizing this elevated incidence. Most fundamentally, the comparison populations in existing literature are not analogous to our cohort. The 3.22% weighted incidence reported by Navarro et al. [11] and the 8–24% range cited in historical series reflect general ADM reconstruction populations, the majority of whom had not received NAC. To our knowledge, no prior study has separately characterized RBS incidence in a homogeneous post-NAC ADM reconstruction cohort; the elevated incidence observed here may therefore reflect a genuine biological vulnerability specific to this population rather than an outlier finding, consistent with the NAC-induced immunologic perturbations described above. Diagnostic and surveillance factors may also contribute: our five pre-specified criteria were applied by two experienced breast surgeons (T.H.K. and H.Y.K.) with complete inter-reviewer agreement on all cases (κ = 1.0), and all patients were followed in a dedicated breast surgery outpatient clinic with systematic postoperative visits, which may capture mild-to-moderate RBS cases that would be missed under less structured surveillance. Finally, ADM product characteristics were also examined; RBS incidence did not differ significantly across the three products used in our cohort (p = 0.780; S1 Table), suggesting that product-specific factors are unlikely to explain the elevated incidence observed in this setting. In aggregate, the elevated incidence most plausibly reflects the convergence of a biologically vulnerable post-NAC patient population and systematic diagnostic ascertainment, rather than product-specific or institutional variation, and should not be interpreted as generalizable to non-NAC ADM reconstruction populations without external validation.

### Novel preoperative predictors: WBC, PLT, and Ki-67 expression

We identified three independent preoperative variables as predictors of RBS: two routine CBC parameters (WBC, PLT) and tumor Ki-67 expression, representing the first systematic demonstration that these readily available preoperative indices can stratify RBS risk in the post-NAC ADM reconstruction population.

The positive association between elevated WBC and RBS (OR 1.235 per ×10³/µL, *p* = 0.004) suggests that a pre-activated systemic inflammatory state at the time of surgery amplifies ADM-triggered immune responses. In the post-NAC setting, an elevated preoperative WBC, paradoxically observed despite chemotherapy-induced myelosuppression, may reflect immune reconstitution with a reactive, pro-inflammatory phenotype. Upon ADM implantation into previously treated tissue, this pre-primed leukocyte milieu may lower the threshold for the lymphatic disruption and immune-mediated cascade underlying RBS [18,21,29].

The positive association between elevated PLT and RBS (OR 1.013 per ×10³/µL, *p* = 0.037) aligns with the well-established role of platelets as critical post-surgical inflammatory regulators. Platelets function not merely as mediators of hemostasis but as active modulators of the inflammatory microenvironment through release of PDGF, TGF-β, and IL-1β from alpha granules [22–24]. Elevated preoperative platelet counts may indicate a hyperactive inflammatory or pro-thromboinflammatory state, potentially amplifying the local immune response to ADM matrix components [22–24]. In the post-NAC context, reactive thrombocytosis, representing an acute-phase response to chemotherapy-induced tissue stress, may identify patients with particularly reactive inflammatory profiles. This is consistent with prior reports demonstrating independent associations between elevated preoperative platelet counts and increased complications in breast reconstruction [25].

The positive association between high Ki-67 expression and RBS (OR 8.975, 95% CI 1.356–155.173, *p* = 0.020) suggests that tumor proliferative activity may serve as a surrogate marker of a heightened baseline immune and inflammatory state. Ki-67 expression correlates with increased stromal tumor-infiltrating lymphocyte density and a more immunologically active tumor microenvironment [26]. We hypothesize that patients with highly proliferative tumors may exhibit a systemic immune profile—potentially mediated by circulating cytokines or activated immune cell populations—that persists into the post-NAC surgical period and predisposes to an exaggerated inflammatory response upon ADM implantation. To our knowledge, no prior study has examined tumor proliferative activity as a risk factor for RBS; existing RBS risk factor analyses have focused exclusively on patient-level (smoking, diabetes, obesity) and surgical/product-level (ADM type, radiotherapy) factors [10,11]. Our identification of Ki-67 expression as an independent correlate of RBS therefore represents a novel mechanistic link between tumor biology and this ADM-specific inflammatory complication. We note that this association carries considerable uncertainty, reflected in the wide confidence interval driven by the small number of Low/Intermediate Ki-67 cases (n = 13); this finding should therefore be regarded as hypothesis-generating and requires independent replication. Importantly, Ki-67 status is routinely determined on the diagnostic core biopsy performed prior to NAC as standard oncologic practice, and therefore does not require any additional testing beyond what is already obtained for every patient undergoing neoadjuvant treatment.

Comparison with prior literature reveals both convergent and divergent findings. Schneider et al. noted associations between inflammatory indices (eosinophils, platelet-to-lymphocyte ratio) and RBS in breast-conserving surgery with diced ADM, but did not develop predictive models [31]. Lee et al. identified NAC as a significant RBS risk factor (OR 4.899) but did not examine specific hematologic parameters [10]. Our study extends this work by demonstrating that within a homogeneous post-NAC ADM population, three specific preoperative variables—individually non-significant (AUC 0.605–0.641, all *p* > 0.10)—achieve clinically meaningful discriminative ability when combined, suggesting that it is the convergent hematologic and tumor-biological phenotype rather than any single parameter that identifies the susceptible patient.

### Clinical implications

Our findings generate several hypotheses with potential clinical relevance, pending prospective validation. First, surgeons and patients considering ADM-assisted immediate reconstruction following NAC should be counseled about the substantially elevated RBS risk of 31.9% observed in our cohort—approximately three- to tenfold higher than the 3.22% weighted incidence reported in recent general ADM reconstruction literature [11]. This risk information is essential for informed shared decision-making and may appropriately influence the choice of reconstruction approach, timing, or surveillance intensity in individual patients.

Second, the hematologic combination score offers a practical preoperative risk stratification tool that requires no additional testing beyond a routine CBC and Ki-67 status, both of which are already obtained as part of standard preoperative and diagnostic assessment. Patients meeting ≥2 of the three criteria (WBC ≥ 9.45 × 10³/µL, PLT ≥ 177 × 10³/µL, high Ki-67 expression)—representing approximately 46.8% of our ADM cohort—constitute a high-risk group in whom enhanced postoperative surveillance, a lower threshold for early anti-inflammatory intervention (corticosteroid therapy), or discussion of alternative reconstruction approaches may warrant further prospective investigation. In particular, patients identified as high-risk should be explicitly counselled on non-ADM reconstructive alternatives, including conventional retropectoral or subpectoral expander-implant reconstruction and selected ADM-free prepectoral techniques, which avoid ADM-associated inflammatory risk entirely [32,33]. Conversely, score-negative patients carry an NPV of 92.0%, providing clinically meaningful reassurance that the large majority will remain RBS-free under standard monitoring protocols.

Third, our findings raise the question of whether a deliberate interval between NAC completion and reconstruction—to allow further immune reconstitution and normalization of WBC and platelet counts—might reduce RBS susceptibility in high-risk patients. Whether the association between tumor proliferative activity (Ki-67) and RBS reflects a modifiable biological pathway, or is instead a fixed marker of underlying tumor and host immune biology, remains to be determined and represents an important direction for translational investigation.

Fourth, our findings suggest that RBS pathophysiology in post-NAC patients may be mechanistically distinct from that in chemotherapy-naïve populations. Notably, neither implant type (*p* = 0.524) nor adjuvant radiotherapy (*p* = 1.000) was associated with RBS development in our cohort—diverging from prior literature identifying radiotherapy as a significant risk factor [10]. This dissociation from traditionally reported surgical and treatment-related determinants, combined with these preoperative hematologic and tumor-biological predictors, points toward chemotherapy-induced immunologic dysregulation and tumor-associated immune activation as central contributing mechanisms rather than reconstruction technique or adjuvant treatment sequence. A clinically important corollary is that the combination score provides risk stratification that is independent of implant type and radiotherapy plan. This reframing may inform future preventive strategies, including investigation of immunomodulatory interventions timed to the post-NAC reconstruction period.

Future research should prioritize external validation in independent, multi-institutional prospective cohorts with standardized RBS diagnostic criteria. Translational studies examining the mechanisms linking hematologic parameters and tumor proliferative activity to ADM-driven inflammation represent a high-priority research direction.

### Limitations

#### Sample size and statistical power.

The primary limitation is the small sample size (n = 47 ADM patients, 15 RBS events; EPV = 5.0). To address this, we replaced standard logistic regression with Firth penalized logistic regression throughout all analyses—a method specifically validated for small-sample binary outcomes that eliminates maximum likelihood bias under sparse data conditions and provides finite estimates even under quasi-complete separation [27,28].

The small sample itself reflects the inherent rarity of the target population. NAC is administered primarily to facilitate tumor downstaging and breast conservation; [1,2] most patients successfully treated with NAC undergo breast-conserving surgery and are excluded from post-mastectomy reconstruction studies. Among the minority requiring mastectomy despite NAC, only a subset qualifies for immediate implant-based reconstruction based on adequate skin envelope and patient preference. Our cohort therefore represents multiple layers of clinical selection—NAC administration, persistent mastectomy indication, and immediate reconstruction suitability—which simultaneously explains the limited sample size and the absence of comparable literature specifically in this population.

#### Model validation and cutoff derivation.

Rigorous bootstrap internal validation (2,000 resamples) yielded a near-zero optimism of −0.001, with an optimism-corrected AUC of 0.817 (95% CI 0.636–0.959) and a stable test AUC distribution across all resamples. The near-zero optimism and stable bootstrap distribution provide empirical support that the combination score’s discriminative performance reflects genuine predictive signal rather than overfitting with respect to the final score’s discrimination. However, bootstrap internal validation was applied to the final composite score after cutoff derivation in the original dataset, and therefore does not fully account for optimism arising from the Youden Index-based cutoff selection process. The reported AUC, sensitivity, and specificity should be regarded as optimistic estimates, and the cutoff values (WBC ≥ 9.45 × 10³/µL, PLT ≥ 177 × 10³/µL, high Ki-67 expression) should be treated as provisional thresholds pending external validation. Furthermore, variable selection instability is an inherent limitation of backward elimination in small-sample settings, and while Firth penalized regression provides coefficient shrinkage, the predictor set identified here should be regarded as provisional and hypothesis-generating. External prospective validation with a pre-specified model will be required to confirm the stability and generalizability of these findings.

#### Extended study period and temporal confounding.

The 14-year study period (January 2011 through May 2025), while necessary to accumulate sufficient cases in this rare population, introduces the potential for temporal confounding through changes in NAC regimens, ADM products, and surgical techniques over time. We directly addressed this concern by performing a formal Cochran–Armitage test for linear trend, which revealed no statistically significant temporal trend in annual RBS incidence (*p* = 0.527) nor in combination score positivity rates (*p* = 0.159) across the ADM study period (2017–2025). The absence of significant trends supports pooled analysis validity. Notably, ADM-assisted reconstruction was introduced at our institution in January 2017, reflecting the broader adoption of ADM techniques in implant-based breast reconstruction over the past decade; consequently, the Non-ADM group includes patients reconstructed before ADM availability, rendering it a partly historical comparator. The Non-ADM/ADM comparison is therefore presented as descriptive context only, not as a causal inference (S4 Table).

#### Outcome ascertainment and diagnostic standardization.

The retrospective design precluded prospective, blinded RBS ascertainment. We addressed this by implementing five pre-specified, operationalized diagnostic criteria adapted from published literature [7,8], given the absence of a universally accepted consensus definition [11]. All records were reviewed by two of three experienced breast surgeons with consensus resolution of discrepancies, and cases not meeting all criteria or with ambiguous documentation were excluded.

#### Other limitations.

Additional limitations include single-center design, which may limit generalizability to institutions using different ADM products, surgical techniques, or NAC protocols. Mastectomy flap thickness — which may vary by location within a single specimen — was not systematically quantified, and intraoperative perfusion assessment was not performed as part of the study protocol; both represent potential unmeasured factors in this retrospective analysis. External validation in independent, prospective cohorts is mandatory before clinical implementation.

Notwithstanding these limitations, the study possesses notable strengths: it is the first analysis specifically focused on RBS prediction in the post-NAC ADM reconstruction population; the cohort’s homogeneity (all patients received NAC, underwent skin-sparing mastectomy, and had immediate implant reconstruction) enhances internal validity; the combination score relies exclusively on routine preoperative laboratory and pathology parameters available in all clinical settings without additional cost; and methodologic rigor—Firth regression, pre-specified diagnostic criteria, and bootstrap validation—provides honest performance estimates.

## Conclusion

In patients undergoing ADM-assisted immediate breast reconstruction after NAC, RBS incidence is substantially elevated (31.9%) and occurs exclusively in the ADM-exposed cohort. Preoperative WBC, PLT, and Ki-67 expression—two routine CBC parameters and a standard pathologic marker already assessed on diagnostic biopsy—are independently associated with RBS, and their combination into a simple scoring rule (≥2 of 3 criteria) achieves AUC = 0.816 (bootstrap-corrected 0.817, 95% CI 0.636–0.959) with sensitivity (86.7%), specificity (71.9%), and NPV (92.0%). To our knowledge, this is the first study to systematically characterize RBS risk and its preoperative determinants in the post-NAC ADM reconstruction population. The provisional combination score—derived entirely from routine preoperative laboratory and pathology parameters requiring no additional testing—may inform future patient counseling, surveillance planning, and prospective study design. External validation in independent prospective cohorts is mandatory before clinical implementation.

## Supporting information

S1 Table Distribution of acellular dermal matrix (ADM) products and red breast syndrome (RBS) incidence by product type in patients undergoing ADM-assisted immediate breast reconstruction after neoadjuvant chemotherapy (N = 47). (ODT)

S2 Table Implant volume in permanent implant recipients stratified by red breast syndrome (RBS) status (n = 14). (ODT)

S3 Table Model calibration, overall performance, and decision curve analysis of the composite hematologic combination score (WBC + PLT + Ki-67 High, cutoff ≥2) for predicting red breast syndrome in patients undergoing ADM-assisted immediate breast reconstruction after neoadjuvant chemotherapy (N = 47). (A) Calibration and overall performance. (B) Confusion matrix at score ≥2 threshold. (C) Decision curve analysis. — Net Benefit by Threshold Probability.
(DOCX)

S4 Table Annual distribution of ADM-assisted and non-ADM reconstruction cases over the study period (January 2011–May 2025). (ODT)
