# Supplementary material for: Predicting red breast syndrome after ADM-assisted breast reconstruction: An exploratory scoring model using routine hematologic markers
Source: PLoS One. 2026 Oct 7;21(10):e0359415. doi: 10.1371/journal.pone.0359415 (PMC13645069; doi:10.1371/journal.pone.0359415)
Supplement: Supplementary file 1 [file pone.0359415.s003.docx]

**S3 Table. Model calibration, overall performance, and decision curve analysis of the composite hematologic combination score (WBC + PLT + Ki-67 High, cutoff ≥2) for predicting red breast syndrome in patients undergoing ADM-assisted immediate breast reconstruction after neoadjuvant chemotherapy (N=47).**

**(A) Calibration and Overall Performance**

| **Metric** | **Value** | **Ideal / Reference** |
| --- | --- | --- |
| Calibration-in-the-large (intercept) | 0.009 | 0.0 |
| Calibration slope | 1.073 | 1.0 |
| Brier score | 0.160 | Lower = better |
| Null model Brier score | 0.217 | — |
| Scaled Brier score (Brier skill score) | 0.262 | Higher = better (max 1.0) |

**(B) Confusion Matrix at Score ≥2 Threshold**

|  | **RBS (Observed +)** | **No RBS (Observed −)** | **Total** |
| --- | --- | --- | --- |
| **Score ≥2 (Predicted +)** | 13 (TP) | 9 (FP) | 22 |
| **Score <2 (Predicted −)** | 2 (FN) | 23 (TN) | 25 |
| **Total** | 15 | 32 | 47 |

*Sensitivity 86.7%, Specificity 71.9%, PPV 59.1%, NPV 92.0% (at observed prevalence 31.9%).*

**(C) Decision Curve Analysis — Net Benefit by Threshold Probability**

| **Threshold probability** | **Model net benefit** | **Treat-all net benefit** | **Treat-none net benefit** |
| --- | --- | --- | --- |
| **0.10** | 0.267 | 0.244 | 0.000 |
| **0.20** | 0.202 | 0.149 | 0.000 |
| **0.30** | 0.113 | 0.027 | 0.000 |
| **0.40** | 0.092 | -0.135 | 0.000 |
| **0.50** | 0.128 | -0.362 | 0.000 |
| **0.60** | 0.117 | -0.702 | 0.000 |
| **0.65** | 0.067 | -0.945 | 0.000 |

*Net benefit values in green indicate that model net benefit exceeds treat-all net benefit at that threshold.*

The combination score provided positive net benefit over both 'treat all' and 'treat none' strategies across threshold probabilities of approximately 0.05-0.65, encompassing the clinically relevant range.

Calibration, Brier score, and decision curve analysis were computed from the continuous predicted probability of the final validated multivariable Firth model (WBC + PLT + Ki-67 High), fitted using the logistf R package (v1.26.1).

Abbreviations: ADM, acellular dermal matrix; RBS, red breast syndrome; WBC, white blood cell; PLT, platelet; TP, true positive; FP, false positive; FN, false negative; TN, true negative; PPV, positive predictive value; NPV, negative predictive value.
