# Supplementary material for: Predicting red breast syndrome after ADM-assisted breast reconstruction: An exploratory scoring model using routine hematologic markers
Source: PLoS One. 2026 Oct 7;21(10):e0359415. doi: 10.1371/journal.pone.0359415 (PMC13645069; doi:10.1371/journal.pone.0359415)
Supplement: Supplementary file 2 [file pone.0359415.s001.odt]

S1 Table. Distribution of acellular dermal matrix (ADM) products and red breast syndrome (RBS) incidence by product type in patients undergoing ADM-assisted immediate breast reconstruction after neoadjuvant chemotherapy (N=47).
ADM Product
N (%)
RBS, n (%)
No RBS, n (%)
p-value
Megaderm® (L&C BIO, Korea)
27 (57.4%)
8 (29.6%)
19 (70.4%)

AlloDerm® (AbbVie, USA)
11 (23.4%)
4 (36.4%)
7 (63.6%)

CGDerm® (CG Bio, Korea)
7 (14.9%)
3 (42.9%)
4 (57.1%)

Total
47 (100%)
15 (31.9%)
32 (68.1%)
0.780
p-value by chi-square test across all three ADM product groups.
All three products are human-derived acellular dermal matrices processed through comparable decellularization methods.
ADM selection was based on institutional availability and surgeon preference.
Abbreviations: ADM, acellular dermal matrix; RBS, red breast syndrome.
