# Supplementary material for: Predicting red breast syndrome after ADM-assisted breast reconstruction: An exploratory scoring model using routine hematologic markers
Source: PLoS One. 2026 Oct 7;21(10):e0359415. doi: 10.1371/journal.pone.0359415 (PMC13645069; doi:10.1371/journal.pone.0359415)
Supplement: Supplementary file 3 [file pone.0359415.s002.odt]

S2 Table. Implant volume in permanent implant recipients stratified by red breast syndrome (RBS) status (n=14).
Variable
RBS (n=6)
No RBS (n=8)
Total (n=14)
Implant volume (cc), mean ± SD
293 ± 75
226 ± 51
255 ± 71
Implant volume (cc), median (IQR)
330 (286–340)
225 (204–240)
245 (218–328)
Range (cc)
135–350
150–330
135–350
p-value*
0.081


* p-value by Mann–Whitney U test.
Analysis restricted to permanent implant recipients (n=18 in ADM group; 3 excluded due to missing volume data, n=1 excluded due to data entry error). Tissue expander patients (n=29) were excluded due to variable fill volumes across the expansion period, precluding direct comparison.
RBS patients showed numerically higher implant volumes compared to No-RBS patients (293±75 cc vs. 226±51 cc); however, this difference did not reach statistical significance (p=0.081), likely reflecting limited statistical power in this small subgroup. Implant type (tissue expander vs. permanent implant) was not significantly associated with RBS in the full ADM cohort (p=0.524; Table 2).
Abbreviations: RBS, red breast syndrome; IQR, interquartile range; SD, standard deviation.
