# Supplementary material for: Predicting red breast syndrome after ADM-assisted breast reconstruction: An exploratory scoring model using routine hematologic markers
Source: PLoS One. 2026 Oct 7;21(10):e0359415. doi: 10.1371/journal.pone.0359415 (PMC13645069; doi:10.1371/journal.pone.0359415)
Supplement: Supplementary file 4 [file pone.0359415.s004.odt]

S4 Table. Annual distribution of ADM-assisted and non-ADM reconstruction cases over the study period (January 2011 – May 2025).
Year
ADM group, n
Non-ADM group, n
Pre-ADM period (2011–2016)
2011
0
4
2012
0
0
2013
0
0
2014
0
4
2015
0
2
2016
0
0
ADM period (2017–2025)*
2017*
1
2
2018
1
0
2019
2
1
2020
4
0
2021
7
3
2022
4
4
2023
10
1
2024
7
1
2025
11
1
Total
47
23
* ADM-assisted reconstruction was introduced at the study institution in January 2017.
Of the 23 Non-ADM patients, 10 (43.5%) underwent reconstruction before ADM introduction (2011–2016) and are therefore historical controls; 13 (56.5%) are contemporaneous with the ADM period (2017–2025).
This distribution reflects the temporal confounding limitation described in the Limitations section. The Non-ADM/ADM comparison is presented as descriptive context only.
Abbreviations: ADM, acellular dermal matrix.
